# Introduction to the special supplement, unlocking the potential of self-injectable contraception: evidence from coordinated investments

**DOI:** 10.1186/s12905-025-04025-2

**Published:** 2025-09-19

**Authors:** Erica Sedlander, Funmilola M. OlaOlorun, Calvin Chiu, Jane Cover

**Affiliations:** 1 Department of Social and Behavioral Sciences, Institute for Health and Aging, University of California, San Francisco, San Francisco, CA United States of America; 2 Department of Community Medicine, College of Medicine, University of Ibadan, Ibadan, Nigeria; 3 PATH, Seattle, WA United States of America

## Background on contraceptive self-injection

Self-injection of contraception, which is made possible by the all-in-one injectable, subcutaneous depot medroxyprogesterone acetate (DMPA-SC), is a novel self-care technology. Given that women can inject themselves at home, it has the potential to circumvent stubborn barriers to contraceptive agency [1]. Some of these barriers include unreliable healthcare access, costs and time required for quarterly clinic visits, low quality of care, provider bias, social stigma of visiting health centers particularly for adolescents and unmarried women, and family or partner disapproval [2, 3]. After receiving training, women self-inject the first time under supervision and are then given take home units to store for future self-administration. Research across sub-Saharan Africa shows that women can feasibly manage this process [4–6]. Furthermore, women who use self-administered DMPA-SC are more likely to continue contraceptive use when compared to those who receive provider-administered DMPA-SC [7–9]. Thus, the option to self-inject may enable more people to use contraception when they want to. However, uptake of self-injection remains low and limited evidence exists on implementation strategies that enable self-injection to reach its full potential.

## Background and objective of the special supplement

As described in the commentary by MacDaniels [10] in this special issue, the Gates Foundation invested in a coordinated effort to understand how self-injection can be best introduced into different markets across six countries (Kenya, Uganda, Nigeria, Malawi, Pakistan, and India). Within this effort, the Gates Foundation sought assistance from the University of California San Francisco (UCSF) to launch the Self-Injection Learning Exchange (SI-LX) with two goals: (1) Aggregate and synthesize grantee learnings and (2) Share resources and facilitate discussions of emergent findings. Made up of twelve different grantees and two funders, the Gates Foundation and the Children’s Investment Fund (CIFF) (who have jointly funded self-injection investments), the SI-LX synthesizes findings and progress across 12 investments into a quarterly slide deck, meets quarterly with funders and grantees, and has organized this special supplement.

The objective of this special supplement is to capitalize on this unique opportunity to reflect across a set of coordinated investments, to bring together diverse and complementary studies on self-injection, and to offer commentary related to lessons learned from this coordinated effort.

## Featured articles in this special supplement

The opening commentary in this issue describes the challenges and lessons learned from funding a coordinated cross-country effort around self-injection from a program officer at the Gates Foundation. MacDaniels [10] also provides background on why the Gates Foundation chose to invest in self-injection as a promising mechanism to expand contraceptive choice among women and girls.

Several articles feature innovative programmatic approaches to enhance women’s training and support for self-injection. Birabwa et al. [11] describe how they used human-centered design to develop a peer-based mentorship program in Uganda that leverages experienced self-injection users to support women’s contraceptive agency in their community as part of the Innovations for Choice and Autonomy (ICAN) project. Rothschild et al., [12] present findings from a mixed-methods evaluation of an empathy-based provider training and community-level awareness intervention in Nigeria (the Delivering Innovation in Self-Care Project – DISC). DISC combines supply and demand side approaches resulting in increased provider confidence and an increase in DMPA-SC service provision. Anglewicz et al. [13] used a nationally representative cross-sectional study to evaluate the DISC project in Uganda and found that self-injection knowledge and attitudes improved with intervention intensity suggesting that programmatic efforts that combine supply and demand side interventions are likely effective.

To help inform programmatic approaches to self-injection, Gitome et al., [14] conducted in-depth interviews with women in Kenya to illuminate their preferences for training approaches on how to self-inject, including a desire to have someone familiar or close to them provide support to navigate the complexities of self-injection beyond the initial training session.

Several papers find that task shifting and task sharing of contraceptive counseling and instruction on DMPA-SC self-injection to lower cadre and community-based providers may improve accessibility and use of self-injection. Cover et al. [15] showed that in Uganda, when provided with appropriate, competency-based training and supervision, community health workers (CHWs) were more likely than clinic health workers to conduct individual training, ensure private time with the client (if trained in a group), show the client a job aid, and advise on disposal. In Nigeria, Akomolafe et al. [16] found that both community pharmacists and patent and proprietary medicine vendors (PPMVs) could feasibly train women to self-inject; women trained at PPMVs (commonly staffed by female nurses, midwives, community health extension workers or community health officers) and community pharmacists found it acceptable.

Several papers evaluate the unique experience of Nigeria, which took a total market approach to scale-up. Using diverse mixed methods including a desk review of existing DMPA-SC implementation and policy documents; data extraction from the Access Collaborative dashboard; and key informant interviews, Afolaranmi et al., [17] provides a landscape assessment of the lessons learned from the scale up of provider-administered and self-injection of DMPA-SC in Nigeria. Furthermore, Challa et al., [18] used mystery client interactions in Nigeria to uncover that fidelity to dispensing and training guidelines differed by facility type. They also found provider bias against offering self-injection for young, unmarried women indicating a need to clarify service provision protocols. Chiu et al., [19] also used mystery clients and in-depth interviews with providers in Nigeria and found that providers stated willingness to dispense self-injection to young, unmarried women did not translate into actual dispensing practices, corroborating the need for additional provider training and awareness. Given the novelty and shift in moving from a provider-administered model to a self-administered model, these papers highlight that training and retraining of providers is essential to address provider biases and ensure clients’ contraceptive counseling and service needs are met.

Two papers developed and validated new short form measures to advance research in the self-care field. Bullington et al., [20] developed and validated a three-item self-efficacy to self-inject scale in Uganda. This tool is a critical contribution to the self-care field; self-reported self-efficacy to enact a behavior is one of the most well-known predictors of doing so [21]. Sedlander et al., [22] also developed and validated a four-item self-injection social norms scale. Using a cohort study, authors also showed that, self-injection social norms (both perceptions that others in your community are self-injecting and that they approve of self-injection) are associated with self-injection interest at baseline and were predictive of self-injecting at follow up. This suggests that supportive social norms around self-injection can contribute to women’s ability to choose this method illuminating a potential area for demand side interventions and the tool to measure it.

Finally, a protocol for a forthcoming study in Pakistan lays out the authors’ plan to conduct a prospective cohort study to compare the 12-month contraceptive continuation rate for women who receive DMPA-SC (initially provider-administered) with that for women who receive intramuscular depot-medroxyprogesterone acetate (DMPA-IM). Authors will also describe how women opting for self-injection of DMPA-SC adhere to standards for commodity storage, injection timing, injection technique, and waste disposal (Tappis et al.,) [23] Given that South Asian markets are relatively new for self-injection, findings from this study will be critical to inform future work.

## Implications for the field

Self-injection of DMPA-SC is a novel contraceptive method and one that requires additional upfront support compared to provider-administered options. As highlighted by MacDaniels [10], the single-method focus of the Gates Foundation’s coordinated investments offers an important opportunity to reflect on the benefits, challenges, unintended consequences, and lessons learned from such a method-specific effort. Within this supplement, some authors such as Birabwa et al. reflect on the complexity and inherent tension in focusing on a single method within an informed choice framework, recommending use of person-centered outcomes like contraceptive agency (the ability to make and act on one’s contraceptive preferences – [24]), rather than contraceptive use or uptake. More work is needed to advance human-rights based measures, including efforts to examine how single method investments can be pursued in ways that reinforce informed choice principles.

Overall, the diversity of approaches included in this supplement underscore the methodological, conceptual, and practical challenges and opportunities for self-injection of contraception. Each paper has significant implications for the field and this supplement could be a helpful road map for researchers and implementors working to improve contraceptive agency with self-injection as one method in the overall contraceptive mix. As several authors point out, self-injection requires a shift in the power dynamic between healthcare provider and patient and requires giving more control to women. As the papers in this special supplement highlight, younger, unmarried women are met with additional barriers. Future work is required to understand equity issues around self-care. For example, who is permitted to do self-care and who is not? And how do we best address issues of provider bias and minimize provider gatekeeping intended to maintain the status quo? This special supplement captures the current and complex state of the field; we hope it will inspire the next generation of self-injection research.

## References

[1] World Health Organization. WHO recommendations on self-care interventions: self-administration of injectable contraception [Internet]. Geneva: World Health Organization. 2020 p. 4. Available from: https://apps.who.int/iris/handle/10665/332332.

[2] Askew I, Wells E. DMPA-SC: an emerging option to increase women’s contraceptive choices. Contraception [Internet]. 2018; Available from: http://www.sciencedirect.com/science/article/pii/S0010782418304025. 10.1016/j.contraception.2018.08.009 30145126

[3] Emily Himes L, Suchman M, Kamanga C, Birabwa S, Gitome E, Omoluabi, et al. Women’s perspectives on the unique benefits and challenges of self-injectable contraception: A four-country in-depth interview study in sub-Saharan Africa. Stud Fam Plann. 2024;55(4):269–90. 10.1111/sifp.12277. https://onlinelibrary.wiley.com/doi/. 39511950 10.1111/sifp.12277 PMC11636776

[4] Burke HM, Packer C, Wando L, Wandiembe SP, Muwereza N, Pradhan S, et al. Adolescent and covert family planning users’ experiences self-injecting contraception in Uganda and Malawi: implications for waste disposal of subcutaneous depot medroxyprogesterone acetate. Reprod Health. 2020;17(1):117. 32746860 10.1186/s12978-020-00964-1 PMC7396890

[5] Cover J, Ba M, Lim J, Drake JK, Daff BM. Evaluating the feasibility and acceptability of self-injection of subcutaneous depot Medroxyprogesterone acetate (DMPA) in senegal: a prospective cohort study. Contraception. 2017;96(3):203–10. 28673645 10.1016/j.contraception.2017.06.010 PMC6381449

[6] Hernandez JH, Akilimali P, Glover A, Emel R, Mwembo A, Bertrand J. Task-shifting the provision of DMPA-SC in the DR Congo: Perspectives from two different groups of providers. Contraception [Internet]; Available from: http://www.sciencedirect.com/science/article/pii/S0010782418302415. 10.1016/j.contraception.2018.07.002 PMC6197837 30031000

[7] Burke HM, Chen M, Buluzi M, Fuchs R, Wevill S, Venkatasubramanian L, et al. Women’s satisfaction, use, storage and disposal of subcutaneous depot medroxyprogesterone acetate (DMPA-SC) during a randomized trial. Contraception. 2018;98(5):418–22. 29758176 10.1016/j.contraception.2018.04.018

[8] Cover J, Namagembe A, Tumusiime J, Nsangi D, Lim J, Nakiganda-Busiku D. Continuation of injectable contraception when self-injected v. administered by a facility-based health worker: A non-randomized, prospective cohort study in Uganda. Contraception [Internet]. 2018; Available from: http://linkinghub.elsevier.com/retrieve/pii/S0010782418301331. 10.1016/j.contraception.2018.03.032 PMC6197833 29654751

[9] Kennedy CE, Yeh PT, Gaffield ML, Brady M, Narasimhan M. Self-administration of injectable contraception: a systematic review and meta-analysis. BMJ Glob Health. 2019;4(2):e001350. 31179026 10.1136/bmjgh-2018-001350 PMC6528768

[10] MacDaniels K. Challenges and opportunities in coordinating investments across countries and channels to expand access to a new contraceptive method. BMC Womens Health. 2025. 10.1186/s12905-025-03949-z. 40903719 10.1186/s12905-025-03949-z PMC12409935

[11] Birabwa C, Phillips B, Amongin D, et al. Development of a community-based peer-support intervention to improve contraceptive agency and diffuse self-injectable contraception in Uganda: application of the human-centered design approach. BMC Womens Health. 2025;25(Suppl 1):110. 10.1186/s12905-025-03614-5. 40065298 10.1186/s12905-025-03614-5 PMC11892169

[12] Rothschild et al. Acceptability and effectiveness of empathy-based provider training and community-level awareness activities on self-injectable contraceptive use in niger, lagos, and Oyo states, nigeria: A mixed methods program evaluation. BMC Womens Health 25 (Suppl 1), (2025). https://bmcwomenshealth.biomedcentral.com/articles/supplements/volume-25-supplement-1. 10.1186/s12905-025-03992-w PMC12421749 40926244

[13] Anglewicz et al. Can a behavior change ‘ecosystem’ increase knowledge of and improve attitudes towards contraceptive self-injection? A nationally representative cross-sectional study assessing the delivering innovation in Self-Care project in Uganda. BMC Womens Health 25 (Suppl 1), (2025). https://bmcwomenshealth.biomedcentral.com/articles/supplements/volume-25-supplement-1.

[14] Gitome S, Suchman L, Okumu S, et al. Knowledge, confidence and social support: Kenyan women’s priority needs for contraceptive self-injection learning through a social cognitive theory lens. BMC Womens Health. 2025;25(Suppl 1):289. 10.1186/s12905-025-03801-4. 40588751 10.1186/s12905-025-03801-4 PMC12207791

[15] Cover J, Namagembe A, Kunihira B, et al. DMPA-SC self-injection experiences of clients and providers in Uganda: the role of community health workers in reproductive self-care service delivery. BMC Womens Health. 2025;25(Suppl 1):341. 10.1186/s12905-025-03850-9. 40640757 10.1186/s12905-025-03850-9 PMC12243150

[16] Akomolafe TO, Okafor EE, Baruwa S, et al. Factors associated with the acceptability of self-injection training by clients receiving DMPA-SC services from community pharmacies and patent and proprietary medicine vendors in Nigeria. BMC Womens Health. 2025;25(Suppl 1):398. 10.1186/s12905-025-03945-3. 40842014 10.1186/s12905-025-03945-3 PMC12369027

[17] Afolaranmi et al. Lessons from the Scale-Up of Provider-Administered and Self-Injection of DMPA-SC in nigeria: A landscape assessment. BMC Womens Health 25 (Suppl 1), (2025). https://bmcwomenshealth.biomedcentral.com/articles/supplements/volume-25-supplement-1. 10.1186/s12905-025-03938-2 PMC12413743 40913231

[18] Challa S, Chiu C, Jegede A, et al. Quality of counseling for self-administering injectable contraception: field evidence from mystery client interactions in Lagos, Nigeria. BMC Womens Health. 2025;25(1):399. 10.1186/s12905-025-03946-2. 40841908 10.1186/s12905-025-03946-2 PMC12369023

[19] Chiu C, Tijani A, Griffith M, et al. How do pro-social tendencies and provider biases affect service delivery? Evidence from the rollout of self-injection of DMPA-SC in Nigeria. BMC Womens Health. 2025;25(Suppl 1):97. 10.1186/s12905-025-03613-6. 40038661 10.1186/s12905-025-03613-6 PMC11877756

[20] Bullington et al. Validation of a measure of contraceptive self-injection self-efficacy in Uganda. BMC Womens Health 25 (Suppl 1), (2025). https://bmcwomenshealth.biomedcentral.com/articles/supplements/volume-25-supplement-1. 10.1186/s12905-025-03982-y PMC12452008 40983923

[21] Bandura A. Health promotion by social cognitive means. Health Educ Behav. 2004;31(2):143–64. 15090118 10.1177/1090198104263660

[22] Sedlander E, Turaga N, Birabwa C, et al. A longitudinal study examining how self-injection social norms are associated with contraceptive self-injectable interest and use in rural Uganda. BMC Womens Health. 2025;25(Suppl 1):288. 10.1186/s12905-025-03878-x. 40588728 10.1186/s12905-025-03878-x PMC12207788

[23] Tappis H. Injectable contraceptive continuation and user experiences in Punjab, Pakistan: a non-randomized prospective cohort study protocol. BMC Womens Health. 2025;25(Suppl 1). https://bmcwomenshealth.biomedcentral.com/articles/supplements/volume-25-supplement-1. 10.1186/s12905-025-03969-9 PMC12416068 40916043

[24] Holt K, Challa S, Alitubeera P, Atuyambe L, Dehlendorf C, Galavotti C et al. Conceptualizing Contraceptive Agency: A Critical Step to Enable Human Rights-Based Family Planning Programs and Measurement. Global Health: Science and Practice [Internet]. 2024; Available from: https://www.ghspjournal.org/content/early/2024/02/12/GHSP-D-23-00299. 10.9745/GHSP-D-23-00299 PMC10906552 38346841

